# Why Close a Bacterial Genome? The Plasmid of *Alteromonas Macleodii* HOT1A3 is a Vector for Inter-Specific Transfer of a Flexible Genomic Island

**DOI:** 10.3389/fmicb.2016.00248

**Published:** 2016-03-08

**Authors:** Eduard Fadeev, Fabio De Pascale, Alessandro Vezzi, Sariel Hübner, Dikla Aharonovich, Daniel Sher

**Affiliations:** ^1^Department of Marine Biology, Leon H. Charney School of Marine Sciences, University of HaifaHaifa, Israel; ^2^Department of Biology and CRIBI Biotechnology Centre, University of PaduaPadova, Italy; ^3^Department of Botany and Biodiversity Research Centre, University of British ColumbiaVancouver, Canada; ^4^The Department of Evolutionary and Environmental Biology, University of HaifaHaifa, Israel

**Keywords:** *Alteromonas*, genomic island, plasmid, genome assembly, metal resistance

## Abstract

Genome sequencing is rapidly becoming a staple technique in environmental and clinical microbiology, yet computational challenges still remain, leading to many draft genomes which are typically fragmented into many contigs. We sequenced and completely assembled the genome of a marine heterotrophic bacterium, *Alteromonas macleodii* HOT1A3, and compared its full genome to several draft genomes obtained using different reference-based and *de novo* methods. In general, the *de novo* assemblies clearly outperformed the reference-based or hybrid ones, covering >99% of the genes and representing essentially all of the gene functions. However, only the fully closed genome (∼4.5 Mbp) allowed us to identify the presence of a large, 148 kbp plasmid, pAM1A3. While HOT1A3 belongs to *A. macleodii*, typically found in surface waters (“surface ecotype”), this plasmid consists of an almost complete flexible genomic island (fGI), containing many genes involved in metal resistance previously identified in the genomes of *Alteromonas mediterranea* (“deep ecotype”). Indeed, similar to *A. mediterranea*, *A. macleodii* HOT1A3 grows at concentrations of zinc, mercury, and copper that are inhibitory for other *A. macleodii* strains. The presence of a plasmid encoding almost an entire fGI suggests that wholesale genomic exchange between heterotrophic marine bacteria belonging to related but ecologically different populations is not uncommon.

## Introduction

The development of next generation sequencing (NGS) technologies has introduced new and much cheaper methods for prokaryotic whole genome sequencing (Quail et al., 2012). In combination with the decreasing price for computational resources needed for bioinformatic analyses, this has resulted in the publication of a large number of new bacterial genomes (Metzker, 2010). More and more “non-bioinformatic” labs are now sequencing their own prokaryotic genomes and facing questions regarding the project such as which sequencing platform to choose, how many libraries should be generated, which assembly method should be used for the libraries, etc. Some of these questions have been addressed in the literature (Loman et al., 2012; Magoc et al., 2013; Ekblom and Wolf, 2014), while others remain unanswered.

One of the main questions facing each new bacterial sequencing project is whether or not the planned use for the genome requires it to be closed into a single, high quality sequence for each DNA molecule (genome or plasmid, defined as “complete” status). The alternative is often a draft genome, which is fragmented into multiple contigs or has many ambiguous bases (‘N’s). For example, if the genome in question is to be compared to the genomes of other organisms to infer the genetic basis for different physiological characteristics or ecological niches, will such a comparison be biased, for example through fragmented representation of function-related genomic regions such as operons, genomic islands or extra-chromosomal elements such as plasmids (Fernández-Gómez et al., 2012)? Notably, as of October 2015 the curated genome database of the National Center for Biotechnology Information (NCBI) contained 49,204 bacterial genomes, yet only 10% of them are defined as “complete genomes”. All the rest are defined as “draft genomes” which range from one contig with many N’s (scaffold) to a large number of contigs whose order is unknown. The difficulty in assembling a closed genome despite the high coverage typically obtained with NGS techniques is usually caused by repetitive sequences including both highly conserved core genes (e.g., ribosomal and tRNA genes) and non-core (accessory) genes which are often harbored on mobile genetic elements such as plasmids and transposons (Loman et al., 2012). Such sequences often produce “break points” in the assembly, resulting in multiple contigs (Hunt et al., 2014). Alternatively, when assembled, such multicopy genes might be collapsed into a single gene sequence (Ekblom and Wolf, 2014), thus missing genetic microdiversity which may be functionally important (Macrander et al., 2015).

Here we present a comparison of four commonly used approaches which we applied for the sequencing and assembly of the medium-sized genome of a heterotrophic marine bacterium, *Alteromonas macleodii* HOT1A3 (Sher et al., 2011): (I) Reference-based assembly using short Illumina reads. In this approach, which is likely the most simple and inexpensive one and can be performed using user-friendly programs with graphic user interface such as Geneious (Kearse et al., 2012), the genome of a closely-related organism is used as a template for assembly. Underlying this approach is the assumption that the reference genome is highly similar to the one being sequenced in gene content and gene order (although this is usually rigorously tested by comparing the generated contigs to the reference). This approach is often used for closely-related strains of pathogenic bacteria (e.g., Mutreja et al., 2011; Loman et al., 2012; Powers et al., 2013); (II) *De novo* assembly using short paired-end reads produced using Illumina platforms. This approach requires more computational power and bioinformatics expertise, but does not rely on a reference genome (see Kisand and Lettieri, 2013; Magoc et al., 2013, for overviews and detailed bioinformatic comparisons of different *de novo* assembly methods). We present results using two different libraries, each assembled using a different program (**Table 1**); (III) A hybrid approach combining the reference-based and *de novo* approaches, aimed at utilizing the *de novo* assembled contigs to close major gaps in the reference-based scaffold. These gaps often contain functionally and ecologically important genetic material (e.g., Genomic islands, Juhas et al., 2009). This approach has been successfully used to assemble the genomes of other strains of *Alteromonas*, our model organism (see below, López-Pérez et al., 2012, 2014); (IV) *De novo* assembly using long sequencing reads produced by the Pacific Biosciences (PacBio) RS II platform (English et al., 2012; Koren et al., 2013). PacBio requires more DNA and of higher quality than Illumina sequencing and relatively advanced computational resources which nevertheless are available on the Amazon Machine Image (http://aws.amazon.com, see details in the materials and methods section).

**Table 1:** General features of all genome assemblies for *Alteromonas macleodii* strain HOT1A3.

Sequencing platform	PacBio RS II	Illumina HiSeq 2000	Illumina GAIIx		
Assembly method	HGAP *de novo*	VELVET *de novo*	MIRA *de novo*	Hybrid assembly	Reference based assembly
Genome size (bp)	Genome 4,652,873 [Plasmid 148,912 ]	4,715,275	4,707,271	4,371,362	4,654,302
Library	399,576 SE long reads	1,810,538 150 bp PE reads	19,859,116 72 bp PE reads		
Mean Illumina library insert size (bp)	-	475 bp	228 bp		
GC%	44.67 [42.3]	44.6	44.63	44.86	44.95
Contigs	1 [1]	122	834	12	114
N50	-	138,989	8891	682,590	80,406
Subsystems	477 [13]	483	470	469	467
CDS	4074 [178]	4178	4893	3796	3679
Hypothetical proteins	946 [95]	1032	1234	824	778
RNAs	87 [0]	80	65	76	68
Complete rDNA operons	5 [0]	0	1	4	5


*^∗^Brackets [] refer to features related to plasmid pAM1A3.*

*^∗∗^All annotation features are based on automatic prokaryotic genome annotation pipeline (RAST, Overbeek et al., 2005).*

*Alteromonas macleodii*, the clade to which our model organism belongs, are opportunistic copiotrophic bacteria belonging to the γ-Proteobacteria, and represent approximately 3–5% of the bacterial population in ocean surface waters (Baumann et al., 1972; Ivanova et al., 2004; Nelson and Wear, 2014). They are highly specialized in adaptation to a wide range of carbon sources, and can be found both free-living and particle associated (on marine snow, biofilms *etc*.; Acinas et al., 1999; García-Martínez et al., 2002). *A. macleodii* are often among the first organisms to respond to perturbations, e.g., during experiments aimed to simulate mixing or DOM addition (McCarren et al., 2010; Shi et al., 2012) and have been suggested to play an important role in carbon cycling (Baker et al., 2013; Nelson and Wear, 2014; Pedler et al., 2014). *A. macleodii* and related strains also affect the growth of other microorganism, inhibiting the growth of several species of algae (Mayali and Azam, 2004) and taking advantage of the dying cells (Sheik et al., 2014). Our specific model organism, *A. macleodii* strain HOT1A3, was selected due its contrasting effect on the growth of two different *Prochlorococcus* strains in laboratory co-culture (Sher et al., 2011).

Until recently the *A. macleodii* strains whose genomes have been sequenced were divided into two major groups, “surface ecotypes” and “deep ecotypes” (López-López et al., 2005). Strains belonging to the surface ecotype have been isolated from many locations around the globe, whereas those belonging to the deep ecotype have all been isolated from the Mediterranean Sea in which the temperature of the deep waters never drops below 12^o^C (López-Pérez et al., 2013). Further phylogenetic analysis of the strains resulted in the re-naming of the “deep ecotype” strains as a new species, *Alteromonas mediterranea* (Ivanova et al., 2015).

While the genomes of these species are highly syntenous (i.e., they share very similar gene organization; Ivars-Martinez et al., 2008), they differ in the gene content of several flexible genomic islands (fGIs). These genomic islands, located at equivalent genomic locations (López-Pérez et al., 2013, 2014), potentially contribute to adaptation and evolution of the organism by gene exchange processes (Klockgether et al., 2004; Ullrich et al., 2005; Rodriguez-Valera et al., 2009; Avrani et al., 2011).

With this background, as we sequenced and assembled the genome of *A. macleodii* HOT1A3, we compared different sequencing and assembly approaches, aiming to identify the costs and benefits of each technique that would provide guidance to our lab and others when embarking on future bacterial genome sequencing projects. Our results show that, compared to the fully closed genome of *Alteromonas* HOT1A3 obtained using PacBio, the genomes assembled *de novo* from short Illumina reads were fragmented but still covered almost all of the genetic data on the full genome. In contrast, using a reference-based assembly resulted in much higher “loss” of genomic data. Nevertheless, the advantage of closing a genome were evident in that, only in this assembly, we could bioinformatically identify the presence of a 148 kbp plasmid, and link this plasmid to almost an entire genomic island previously observed exclusively in the genomes of *A. mediterranea* strains.

## Materials and Methods

### *Alteromonas Macleodii* HOT1A3 Isolation and Maintenance

*Alteromonas* HOT1A3 was previously isolated in 2007 from a water sample collected at the HOT station ALOHA (22^o^45′ N 158^o^ W) at a depth of 5 m during the C-More BLOOMER cruise on August 18, 2007 (Sher et al., 2011). The water sample was transported back to the Chisolm lab at MIT and maintained for 1 month in clear polycarbonate bottles at 21^o^C under 18 μE constant cold white illumination. The strain was isolated by plating on solid ProMM plates followed by six rounds of streaking and colony picking on the same media. ProMM is based on the Pro99 media used to culture *Prochlorococcus* (Moore et al., 2007), with the addition of a set of defined organic compounds (0.05% w/v each of Lactate, Pyruvate, Acetate and Glycerol) and vitamins. The isolate was initially identified as *A. macleodii* based on the sequence analysis of 16s rRNA gene fragment. The strain is routinely cultured in ProMM media and maintained as frozen stocks at -80°C in 25% (v/v) glycerol in ProMM.

### DNA Extraction and Sequencing

DNA was extracted using the phenol chloroform protocol (Neumann et al., 1992). The genome was sequenced separately in three sequencing runs: (1) Using an Illumina GAIIx platform (72 bp paired-end read). The libraries for this run were produced as follows: DNA was sheared by sonication using a Bio-Ruptor ultrasonicator (Diagenode), end-repaired using the Quick Blunting kit from New England Biolabs, and Illumina adaptors were ligated using the quick ligate kit from Enzymatics followed by PCR enrichment. Size selection was performed using SPRI beads to obtain fragments with a mean size of 228 bp; (2) Using an Illumina HiSeq2000 platform (150 bp paired-end read). The libraries for this sequencing run were produced at the University of Haifa Bioinformatics Support Unit using the NEBNext Ultra DNA Library Prep Kit for Illumina followed by size selection using SPRI beads to obtain fragments with a mean size of 475 bp; (3) Using a Pacific Biosciences RS II platform, with the libraries produced by the DNA template prep kit 3.0 from Pacific Biosciences, at the Yale Center for Genome Analysis.

### Genome Assembly

Each of the different genome assemblies was obtained using a combination of sequencing libraries and assembly programs ad shown in **Table 1**: (1) The Illumina GA library was assembled *de novo* using MIRA short-reads assembler version 3.4.0 with default parameters (Chevreux et al., 2004). In addition, we attempted to assemble the genome using a reference genome of closely related strain. We chose *A. macleodii* ATCC27126 strain as a reference due to the highest percentage of mapped reads and the highest coverage across the entire genome, compared to other published genomes of *A. macleodii*. In order to close the gaps in the produced scaffold, we mapped the contigs generated by MIRA to the reference-based assembly using the reference mapping function of Geneious 7 (Kearse et al., 2012), followed by manual validation of the results (termed as “hybrid assembly”). (2) The Illumina HiSeq2000 *de novo* assembly was obtained using a custom pipeline. The initial assembly was performed using VELVET assembler (v. 1.2.10; Zerbino and Birney, 2008) with a *k*-mer size of 81 bases, considering the reads as paired. The assembly was evaluated using the REAPR tool (v. 1.0.16) with standard parameters (Hunt et al., 2013). The resulting corrected contigs were arranged into scaffolds using SSPACE scaffolder algorithm (v. 3.0) with default options (Boetzer et al., 2011). Additional evaluation using REAPR was performed to make sure that no mis-assembly was introduced. Gaps within the scaffolds were filled using GapFiller (v. 1.10; Boetzer and Pirovano, 2012), specifying the following options -m 80 and -o 5 (these option specify for: m, the minimum number of overlapping bases with the sequences around the gap, and o the minimum number of reads needed to call a base during an extension). As final step after the gap filling procedure we ran REAPR to assess that the final assembly was consistent and no error was introduced by GapFiller. (3) The PacBio library was assembled using the Hierarchical Genome Assembly Process (HGAP, Chin et al., 2013), integrated in the SMRT analysis software (version 2.3.0) from Pacific Biosciences. The contigs were closed into circular molecules by alignment of 10 kbp from both ends of the contig to each other, using the Geneious 7 alignment tool (Kearse et al., 2012).

### Gene Prediction and Annotation

All assemblies were annotated using Rapid Annotation using Subsystem Technology (RAST) prokaryotic genome annotation server (Aziz et al., 2008; Brettin et al., 2015). The genome and plasmid generated by PacBio sequencing were additionally annotated based on all published genomes of *A. macleodii* in NCBI genome database (NC_011138.3, NC_018632.1, NC_018678.1, NC_018692.1, NC_018679.1, NC_019393.1, NC_023045.1, NC_021717.1, NC_021712.1, NC_021713.1, NC_021714.1, CP004846.1, CP004849.1) using Prokka (version 1.10) genome annotation tool (Seemann, 2014). Both annotations were merged together by comparison of location of the genes (coordinates on the genome). The final annotation consisted of 4215 genes in the genome and 184 genes in the plasmid. Gene ontology (GO) terms were assigned to the CDS using InterProScan5 (Jones et al., 2014). In addition, due to the discovery of several metal resistance related operons on the plasmid, the annotation of all its genes was verified using BLASTP (Moreno-Hagelsieb and Latimer, 2008) to BacMet dataset – a high quality, manually curated database of experimentally confirmed antibacterial biocide- and metal-resistance genes (Pal et al., 2014). All genomic maps of genome and plasmid annotations and comparisons were generated using BRIG visualization tool (Alikhan et al., 2011) and Circos visualization tool (Krzywinski et al., 2009).

### Comparison of Genome Assembly Products

In order to assess the quality of each one of the assembly approaches we used, the products of each assembly was compared to the final genome and plasmid sequences. Genome fraction calculations of each assembly method was conducted using the QUality ASsessment Tool for genome assembly (Gurevich et al., 2013). The number of fully contained proteins in the contigs of the fragmented assemblies was calculated aligning all the annotated proteins from the final genome and plasmid using tBLASTn to all ORFs of each one of other assemblies, where only completely aligned proteins were counted (Magoc et al., 2013). A statistical comparison between the assemblies was conducted using Fischer’s exact test of gene counts assigned to different subsystems according to SEED categorization system, which organizes gene functional category into 5 hierarchical levels (Overbeek et al., 2005).

### Comparison of *Alteromonas* HOT1A3 to published *A. macleodii* Genomes

All published genomes of *A. macleodii* were re-annotated using RAST annotation pipeline. Using BLASTn alignment (coverage of at least 80% of the total length of the gene with an *e*-value ≤ 0.0001) we detected the core genes which were present in all the genomes, as well as “surface ecotype” and “deep ecotype” related genes. All alignments were conducted using Geneious 7 aligner (Kearse et al., 2012) and phylogenetic trees were generated using MEGA6 software (Tamura et al., 2013). Functional comparison between the genomes was conducted using principal component analysis of gene counts assigned to different subsystems according to SEED categorization system.

### Pulse Field Gel Electrophoresis

To validate the presence of the bioinformatically-identified pAM1A3 plasmid, pulse field gel electrophoresis (PFGE) was carried out (Hightower et al., 1987; Mathew et al., 1988). In order to extract only the plasmid DNA, DNA was extracted using the NucleoBond^®^ BAC 100 kit (Blaas et al., 2012). In order to verify the actual size of the plasmid, it was cut using three different restriction enzymes: NotI, SgrAI, PacI (Barth et al., 2009). PFGE of the extracted DNA was performed using CHEF DRIII device with 1% agarose gels and 0.5X TBE buffer at 14^o^C for 11.5 h. Lambda ladder PFGE marker, low range ladder PFGE marker and 1 KB DNA ladder marker were used as molecular size markers. The gel was stained using GelRed stain (Biotum, Hayward, CA 94545) and photographed over an UV transilluminator (López-Pérez et al., 2012; Marasini and Fakhr, 2014; Ozdemir and Acar, 2014).

### Heavy Metal Resistance Assays

Heavy metal resistance was assessed by growth experiments in ProMM medium with heavy metals (mercury chloride, zinc acetate, or copper chloride) added at different concentrations (0.1, 0.25, 0.5, 0.75, 1 nM for zinc and copper and 0.001, 0.01, 0.05, 0.1 mM for mercury). The cultures were grown in 96 well plates at 20 ± 2°C inside a Perkin-Elmer Enspire multimode plate reader with the optical density at 600 nm measured every 1 h. The minimal inhibitory concentration was defined as the concentration where no growth was observed after 36 h.

### Accession Numbers

The sequences have been deposited in GenBank under the genomic accession numbers CP012202 for the *A. macleodii* HOT1A3 genome and CP0122203 for the plasmid pAM1A3.

## Results

### The Fully Assembled Genome of *A. macleodii* HOT1A3

**FIGURE 1:**
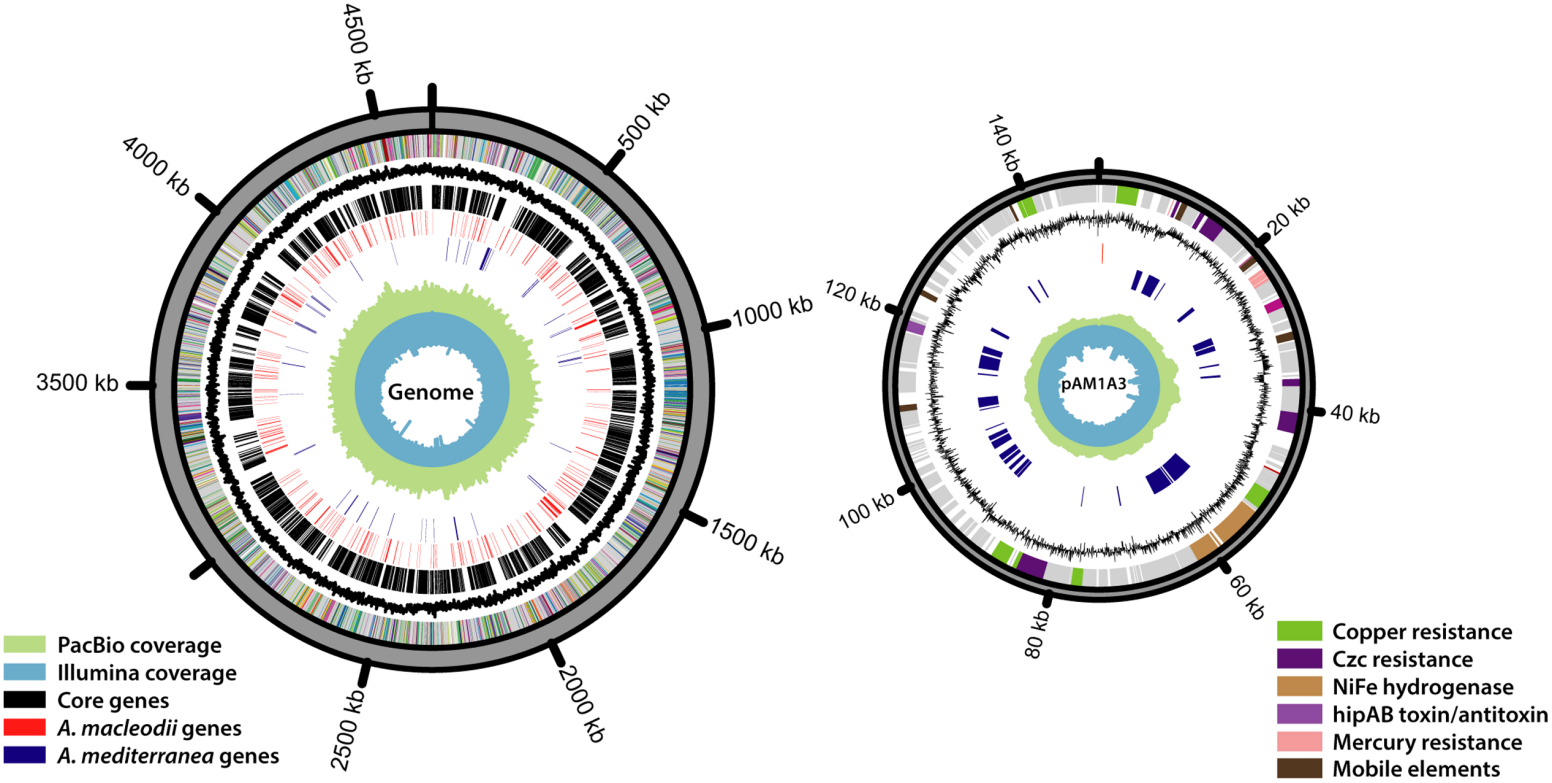
**The genome of *Alteromonas* HOT1A3 and its plasmid, pAM1A3.** The rings, ordered from the inside out, represent the following data: Illumina read coverage, PacBio read coverage, *A. mediterranea* specific genes (blue), *A. macleodii* specific genes (red), *Alteromonas* core genes (black), GC contents, different subsystems assignment of the genes. Genes with no functional annotation are colored gray.

Aiming to obtain a completely closed genome for *Alteromonas* HOT1A3, we conducted several assembly attempts using different DNA-seq libraries and different assembly approaches (**Table 1**). The assembly of the PacBio library generated two contigs, sized 4.6 Mbp and 148 kbp, which we could close into two circular DNA molecules (**Figure 1**). Pulse-field gel electrophoresis combined with restriction enzyme analysis revealed the presence of two closely-related plasmids, differing by about 6 kbp (**Supplementary Figure S1**). Two regions in the plasmid exhibited increased sequencing coverage and a high number of single nucleotide polymorphisms (SNPs), suggesting that these regions are duplicated, potentially in tandem, on some of the plasmids (**Supplementary Figure S1**). The number of total SNPs identified in the sequences was 697 for the genome and 65 for the plasmid, attesting to the high quality and homogeneity of the genome. The genome too had a region of ∼70 kbp with approximately double coverage, which did not have a higher density of SNPs compared to the rest of the genome. This region may also represent a tandem repeat.

**FIGURE 2:**
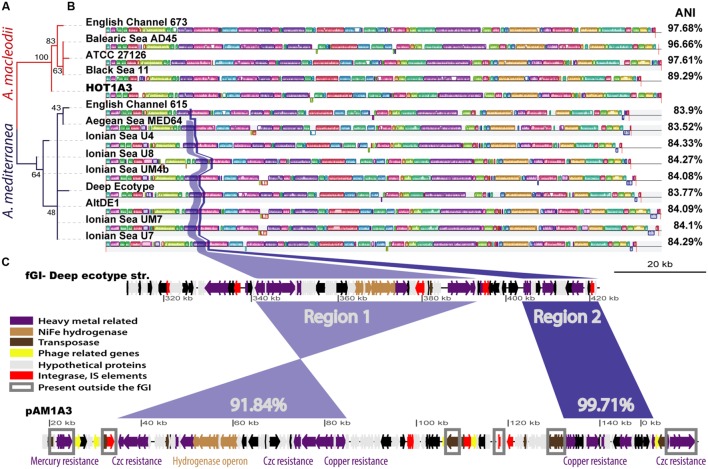
**An overview of the similarity between the genome of HOT1A3 and its plasmid and that of other *Alteromonas* strains.** **(A)** Maximum Likelihood tree of all 16S rDNA sequences of *Alteromonas* strains for which a whole genome sequence is available. Red branches refer to *A. macleodii* and dark blue branches to *A. mediterranea*. The numbers on the branches represent bootstraps percentage. **(B)** Whole genome alignment of all the strains, produced by MAUVE (Rissman et al., 2009), with the HOT1A3 genome marked in red. The order of the genomes corresponds to that of the phylogenetic tree in **(A)**. The differently colored blocks along the genomes represent collinear genomic regions or locally collinear blocks (LCBs, Darling et al., 2011). The average nucleotide identity (ANI) percentage values refer to comparison between the genome of HOT1A3 and each one of the strains. Two regions of high similarity between flexible genomic island (fGI) 2 of *A. mediterranea* and the plasmid pAM1A3 are highlighted in blue and purple. **(C)** Representation of flexible genomic Island 2 of *A. mediterranea* type strain DE and the plasmid pAM1A3. The genes are colored based on functional categories (see legend). The percent numbers refer to pairwise nucleotide identity between the regions. Genes marked with gray rectangles are present in other regions of several *A. macleodii* and *A. mediterranea* genomes.

Using the 16S rDNA gene as a phylogenetic marker, *Alteromonas* HOT1A3 belongs to the *A. macleodii* (**Figure 2A**). This was supported by a phylogenetic analysis of eleven concatenated core genes (**Supplementary Figure S2**) as well as by whole-genome comparison, revealing that the genome of HOT1A3 has 97% nucleotide identity with the genome of *A. macleodii* ATCC 27126, which is considered to be the type strain for *A. macleodii* (**Figure 2B**). Notably, these two strains were both isolated near Hawaii >35 years apart (Baumann et al., 1972; Sher et al., 2011). Finally, the classification of *Alteromonas* HOT1A3 as belonging to the *A. macleodii* species rather than to *A. mediterranea* was also evident by the number of genes shared by *Alteromonas* HOT1A3 with one of these species but not the other (see red and blue gene layers in **Figure 1**). We use the fully assembled genome, produced from PacBio reads, as a benchmark to assess the results of all other assembly methods.

### Comparison of the Genomes Obtained Using Different Assembly Methods

The predicted genome size of the various *de novo* assemblies was quite close to that of the closed genome with its plasmid, despite their fragmentation into hundreds of contigs (**Table 1**). In contrast, the reference-based assembly and the hybrid assembly both predicted somewhat smaller genomes. Of all the fragmented assemblies, the one produced using longer Illumina reads with a larger insert size, and assembled *de novo* using VELVET, had the closest number of genomic features (coding sequences [CDSs], hypothetical proteins and RNAs) to the final PacBio-sequenced genome and plasmid (**Table 1**). However, the fragmented nature of this assembly resulted in it not containing completely assembled ribosomal operons.

**FIGURE 3:**
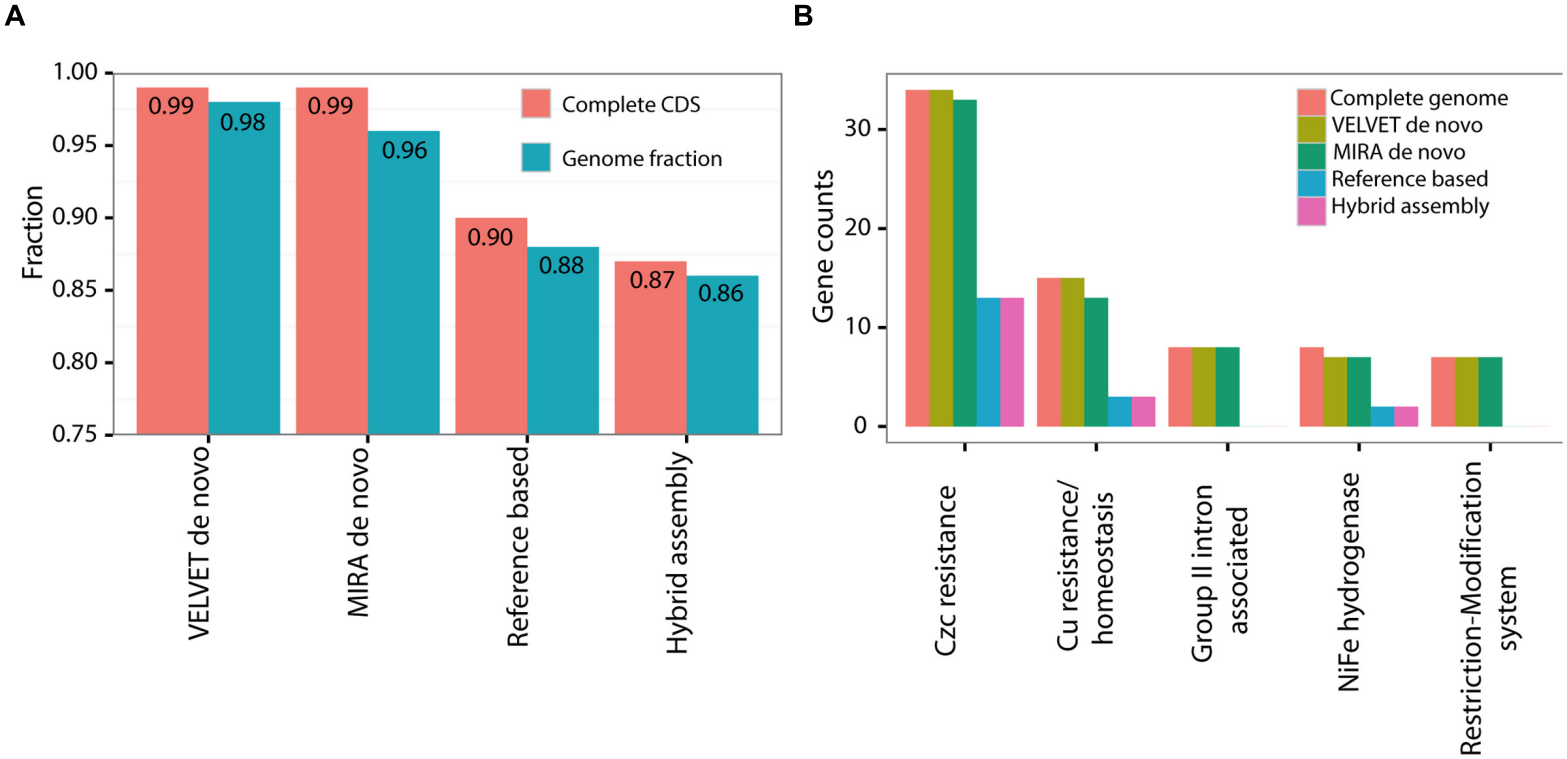
**A comparison of the various assembly methods to the complete genome of HOT1A3 strain.** **(A)** Nucleotide sequence length and number of fully covered CDSs. **(B)** Gene counts within the five significantly different subcategories. The comparison of 100 most abundant functional SEED subsytems, used to calculate the significance of the results, is shown in **Supplementary Figure S3**.

We next assessed what specific and functional genetic information had been lost in each of the draft genomes compared to the closed PacBio genome. We used three metrics for this comparison: a) the length of the nucleotide sequence of the genome and plasmid combined (a total of 4.8 Mbp, **Figure 3A**); (b) the number of complete predicted genes (Coding DNA Sequences or CDSs, **Figure 3A**); (c) the representation of functional pathways, defined using the SEED subsystems approach (**Figure 3B**; Aziz et al., 2008). The *de novo* assemblies clearly outperformed the hybrid and reference-based ones, covering almost the entire genome both in terms of nucleotide sequence and in terms of CDSs (**Figure 3A**). In all of the assemblies the fraction of complete CDSs was higher than the fraction of the nucleotide sequence itself, suggesting that at least some of the sequences hindering assembly are found in intergenic regions. While the representation of most of the functional groups was very similar between the different assemblies, there were five functional subsystems which were significantly underrepresented within the reference based assemblies but not in the *de novo* assemblies (**Figure 3B**, **Supplementary Figure S3**, Fisher’s Exact Test, *p* = 0.05). The missing genes in these subsystems are involved in metal resistance, hydrogenase activity and RNA metabolism, and the majority of them are found on the plasmid (see below). Thus, while the *de novo* assemblies capture almost all of the genes and pathways present in the closed genome, the reference-based and hybrid assemblies had reduced representation of several potentially important genes and pathways. This could lead to biases or errors in attempting to interpret how the functions encoded in the genome relate to the organism’s life history or ecology, especially in reference to the effect of heavy metals.

### *Alteromonas* HOT1A3 Hosts a Plasmid – pAM1A3

One unique genomic feature, which emerged from the closed genome but not from the draft ones, was the presence in *Alteromonas* HOT1A3 of a plasmid. The plasmid, termed pAM1A3, is ∼148 kbp long and encodes 184 genes (**Figure 1**). Among these genes there are several plasmid-related features such as genes encoding the bacterial *parA*- partitioning protein, which ensures proper distribution of plasmid replicates to daughter cells during cell division (Bignell and Thomas, 2001) and the methylation subunit of a restriction modification system which may protect the plasmid from specific restriction enzymes (Tock and Dryden, 2005). The plasmid also encodes a putative *hipAB* toxin-antitoxin gene pair, which consists of two genes, *hipA* which encodes a stable toxin and *hipB* which encodes an unstable antitoxin in form of a repressor for the *hipAB* operon (Wen et al., 2014). This mechanism develops an “addiction” of the cell by constant need of antitoxin expression, and may represent a mechanism for the maintenance of the plasmid despite the related metabolic cost (Yamaguchi et al., 2011). The gene *hipA* was not found in any of the *A. macleodii* published genomes, whereas a *hipB*-like gene is found on a plasmid which was isolated from *A. mediterranea* AltDE1 strain (Gonzaga et al., 2012). The plasmid does not contain genes involved in conjugation, but many genes involved in the biosynthesis of type IV secretion systems (pili) are found on the genome.

There are several transcriptional regulators found on the plasmid, among them member of the *marR* family of regulatory proteins, which are known to regulate the expression of multidrug efflux pumps (Nikaido, 1998; Alekshun et al., 2001), and three copies of *merR*- family transcriptional regulator, which is a common transcription regulation system often associated with plasmids (Silver and Walderhaug, 1992).

Among the functional genes encoded on the plasmid, many belong to several operons associated with metal resistance (**Figure 2C**). The mercury resistance operon *merRTPCA*, includes the regulatory protein *merR*, and three mercury transport proteins *merT*, *merP*, and *merC* (Summers, 1992). The operon found on the plasmid also contains *merA*, a key enzyme with a role in mercury detoxification, yet lacks the lyase subunit *merB* which may be necessary for the activity of *merA* (Huang et al., 1999). The plasmid also encodes the copper resistance genes *copABCDG*, as well as a two-component regulatory module *cusRS*, which typically regulates the cop operon (Grass and Rensing, 2001; Bouzat and Hoostal, 2013). Finally, there are two operons of cobalt-zinc-cadmium resistance (*czcABC*) which encode the components of heavy metal efflux pumps (Silver and Walderhaug, 1992).

In addition to the heavy metal resistance operons, the plasmid also hosts a complete gene cluster encoding a *NiFE* hydrogenase complex which is completely absent from all published *A. macleodii* genomes, with an exception of the draft genome of *A. macleodii* MIT1002 (Weyman et al., 2011; Biller et al., 2015). pAM1A3 also has several transposable elements which are associated with horizontal gene transfer such as Tn3-related resolvase and transposase genes, IS66- family and Tn21-family transposases (Liebert et al., 1999; Han et al., 2001; Parks and Peters, 2007).

### Plasmid pAM1A3 Contains Almost an Entire Flexible Genomic Island

Some *A. macleodii* and *A. mediterranea* strains harbor plasmids, however, as shown in **Supplementary Figure S4**, none of these plasmids revealed high similarity to pAM1A3, with an exception of a small region of 7 kbp which encode Tn3-related resolvase and transposase genes. Instead, more than half of the functional genes on the pAM1A3 plasmid have close homologs in all the genomes of *A. mediterranea*, and almost no representation in the genomes of *A. macleodii* strains. These functional genes, which include many of the metal resistance genes as well as the hydrogenase genes described above, belong to two almost contiguous regions (**Figure 2** and **Supplementary Figure S4**), both of which correspond to a fGI previously identified in strains belonging to the *A. mediterranea* species (described as the “Deep ecotype” in **Figure 2C**; Ivars-Martinez et al., 2008; Gonzaga et al., 2012) and in one of two strains of *A. australica* (López-Pérez et al., 2014). Remarkably, a similar genomic island was also found on a plasmid hosted by *Glaciecola* sp. strain 4H-3-7 + YE-5 (pGLAAG01, **Supplementary Figure S5**, Klippel et al., 2011; López-Pérez et al., 2014).

In terms of its gene content and organization, the region encoding these genes on the plasmid is closest to fGI-2 of the *A. mediterranea* type strain DE, containing ∼75% of the genes (∼80% of the fGI sequence) and revealing ∼91.8–99.7% nucleotide sequence identity (**Figure 2C**). Thus, it is tempting to speculate that the genes on the plasmid were acquired almost wholesale from a strain closely related to DE strain. Nevertheless, phylogenetic analysis of several of the genes on the plasmid, e.g., *copA*, *merR*, and *czcA*, showed that the phylogeny of the genes on the plasmid is not homogenous, and that some are phylogenetically closer to other strains of *A. mediterranea* and not to DE (**Supplementary Figure S6**). It is currently unclear whether this reflects the evolutionary history of the fGI on this hypothetical donor strain or whether the acquisition of novel genetic information and/or its rearrangement occurred on the plasmid.

**FIGURE 4:**
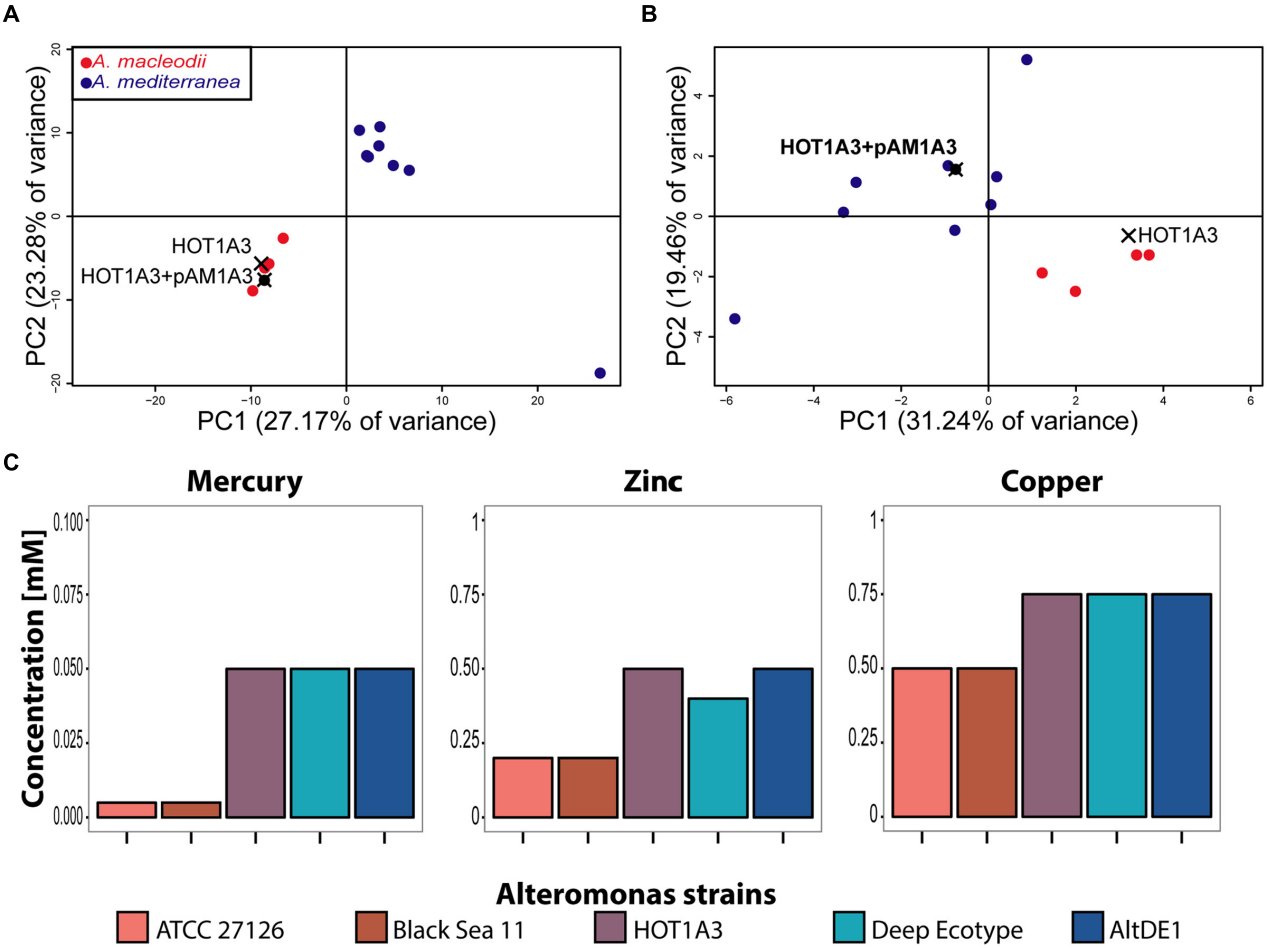
**The presence of the plasmid pAM1A3 may affect the functional genetic capacity of *Alteromonas macleodii* HOT1A3.** **(A,B)** Principal Component Analysis (PCA) of the functional capacity of genomes of *A. macleodii* and *A. mediterranea* strains, defined as the presence and number of genes belonging to specific SEED subsystems (Overbeek et al., 2005). **(A)** Shows the results when all of the subsystems found on the genome and plasmid are considered, whereas **(B)** shows the results when only the subsystems found on the plasmid (including genes belonging to these subsystems on the genome) are considered. Note that on this panel HOT1A3 is shown twice, once with and once without the plasmid, to show the impact of the plasmid on these functional categories. **(C)** Minimal inhibitory concentration of heavy metals in growth assays of three *A. macleodii* (ATCC 27126, Black Sea 11 and HOT1A3) and two *A. mediterranea* (Deep Ecotype, AltDE1) strains.

Given that, with its plasmid, *Alteromonas* HOT1A3 has a mixture of features associated with *A. macleodii* and *A. mediterranea*, we asked to which of these groups strain *Alteromonas* HOT1A3 is more closely related in terms of functional metabolic capacity. We re-annotated the genomes of the previously described *A. macleodii* and *A. mediterranea* strains using the same pipeline we used for *Alteromonas* HOT1A3, and performed a principle component analysis (PCA) of the genomes, based on their functional capacity (the SEED subsystems, see Materials and Methods). As shown in **Figure 4A**, a strong clustering of the genomes into two distinct groups is observed, one containing *A. mediterranea* strains and one containing *A. macleodii* strains. In this clustering *Alteromonas* HOT1A3 clearly partitions with *A. macleodii*, in agreement with the phylogenetic analysis and the genome-wide comparisons (**Figures 1** and **2**). However, when the PCA was performed using only the subsystems associated with the plasmid, *Alteromonas* HOT1A3 clustered with the *A. mediterranea* (**Figure 4B**). This suggests that, while clearly belonging to the *A. macleodii* species, for some specific functional features, e.g., metal resistance, *A. macleodii* HOT1A3 may have acquired through its plasmid some of the functional potential of *A. mediterranea* strain.

To assess this hypothesis, we grew representative strains of both *A. macleodii* (ATCC 27126 and Black Sea 11 – BS11; López-Pérez et al., 2012) and *A. mediterranea* (DE, AltDE1; Gonzaga et al., 2012) in ProMM media with different concentrations of heavy metals (zinc, mercury, and copper). As shown in **Figure 4C**, the two *A. macleodii* strains, ATCC 21726 and BS11 were more sensitive to heavy metals (a lower minimum inhibitory concentrations, MIC) than the *A. mediterranea* strains, in agreement with previously published metal resistance comparison (Ivars-Martinez et al., 2008). In contrast, *A. macleodii* HOT1A3, which contains the plasmid with the metal resistance genes, was as resistant to the heavy metals as the *A. mediterranea* strains, supporting the hypothesis that the metal resistance genes on the plasmid are functional.

## Discussion

Horizontal gene exchange is of importance in the adaptation and evolution of bacteria, and the involvement of plasmids in exchange of genetic material and shaping of fGIs in pathogenic bacteria is well recognized (recently reviewed by Juhas et al., 2009; Morales-Espinosa et al., 2012). In marine bacteria, the role of phage-mediated lateral gene transfer in shaping bacterial genomes has been extensively explored (reviewed by Rodriguez-Valera et al., 2009; Rohwer and Thurber, 2009; Breitbart, 2012), perhaps due to the expectation that in dilute seawater transduction, rather than conjugation, is a more likely form of genetic exchange. Nevertheless, the presence of plasmids has long been recognized in marine bacteria (Sizemore and Colwell, 1977), and they are involved in ecologically-important aspects such as growth under low iron conditions, adherence of heterotrophic bacteria to algae and pathogenicity (e.g., Frank et al., 2015; Lee et al., 2015; Osorio et al., 2015). Additionally, the exchange of plasmids via conjugation has been demonstrated in batch seawater (e.g., Dahlberg et al., 1998). In this regard, previous studies have suggested that many of the genomic islands in *A. macleodii* and *A. mediterranea*, and especially the metal resistance related island fGI2, are constantly being re-shaped by local insertion and deletion events, and are thus termed “additive” (Gonzaga et al., 2012). Our observation of a plasmid, pAM1A3, that contains almost this entire genomic island, suggests an alternative mechanism, whereby large amounts of genetic material are transferred wholesale via plasmids. Indeed, previous studies of the genomes of *A. macleodii* and *A. mediterranea* strains have suggested plasmid mediated insertions of three genomic regions, two relatively small (19–21 kbp) and one large (65 kbp), the latter encoding a hybrid polyketide/non-ribosomal peptide synthases gene cluster (López-Pérez et al., 2013). Kalhoefer et al. (2011) recently observed a similar case in a different group of marine bacteria, whereby a relatively large plasmid (∼94 kbp) from *Roseobacter litoralis* was highly similar to a genomic region from a related strain, *R. denitrificans* (Kalhoefer et al., 2011). This suggests that plasmid-mediated gene exchange, including transfer of large amounts of genetic material from the genomes into plasmids and vise-versa, is common in at least some linages of heterotrophic marine bacteria. Whether such genetic exchange occurs when the cells are free-living (planktonic) or particle associated is currently unknown.

How common is the presence of a pAM1A3-like plasmid, with the genetic information it encodes, in Alteromonads in nature? Previously, it has been shown that the global ocean survey (GOS) metagenome contains many sequences related to the metal resistance genes found in the metal resistance fGI, and in fact these genes were more common than the core genes of this strain (Ivars-Martinez et al., 2008). Recently, another *A. macleodii* strain, MIT1002, has been shown to contain some genes found in *A. mediterranea*, including metal resistance genes and a hydrogenase (Biller et al., 2015). Indeed, the genome of this strain contains a single 70 kbp contig with almost complete representation of region 1 of the pAM1A3 plasmid, as well as an additional contig which consists of 12 kbp of pAM1A3 region 2 (**Supplementary Figure S7**). Due to the fragmented nature of the draft genome assembly of MIT1002 it is currently unclear whether this strain contains a plasmid or whether the relevant genes have been integrated into its genome. *Alteromonas macleodii* strain MIT1002 originated from an enrichment culture of *Prochlorococcus* NATL2A collected in 1990 from the North Atlantic, but was physically isolated from it only 20 years later (Biller et al., 2015). Strain HOT1A3 was isolated from the Pacific Ocean near Hawaii in 2007 (Sher et al., 2011). Both of the strains originate from surface waters. Taken together, the GOS metagenomic results, the presence of *A. mediterranea* related genes in *A. macleodii* strains likely originating from two separate oceans and isolated almost two decades apart, and the presence of similar genes in *A. australica* and in a plasmid from *Glaciecola* sp., suggest a global distribution of genes involved in heavy metal resistance and hydrogenase activity among the *Alteromonadaceae*. It also suggests that plasmids such as pAM1A3 are likely vectors for genetic transfer of such information among distantly related *Alteromonas* species or even among *Alteromonadaceae* genera.

Maintaining extra-chromosomal amplicons, such as plasmids, incurs significant metabolic cost (Platt et al., 2012). What advantage does *Alteromonas macleodii* HOT1A3 gain, if at all, by maintaining the pAM1A3 plasmid? Similar to other plasmids (Cytryn et al., 2008; Kalhoefer et al., 2011), pAM1A3 encodes many metal resistance genes, and these may be responsible for its increased resistance to Zn, Hg, and Cu compared to other *A. macleodii* strains (**Figure 4**). The oceanographic profiles of many heavy metals are similar to those of nutrients such as nitrate and phosphate, with maxima in deep waters (Bruland and Lohan, 2003; Morel et al., 2003). The concentrations of Zn, Hg, and Cu in deep waters are several orders of magnitude below the MIC we measured (up to 10 nM for Zn, e.g., in the North Pacific, Bruland et al., 2014, compared to a MIC of 200 nM, **Figure 4**), and most of these metal ions are complexed to organic compounds and not directly bioavailable. Nevertheless, even very low levels of heavy metals (140-fold lower than the MIC) have been shown to sufficiently affect bacterial physiology to offset the maintenance cost of a plasmid with a similar size to that of pAM1A3 (Gullberg et al., 2014). Additionally, heavy metal concentrations may be significantly higher on particles such as dust or zooplankton (e.g., Wang, 2002), and as copiotrophic bacteria *Alteromonas macleodii* and *A*. *mediterranea* have both been suggested to colonize such particles (Acinas et al., 1999; García-Martínez et al., 2002; Ivars-Martinez et al., 2008). Alternatively, other genes on the plasmid may be providing some fitness advantage, such as the hydrogenase operon (Vargas et al., 2011; Weyman et al., 2011) or any of the other >150 genes on the plasmid, most of which have unknown functions. Finally, the plasmid may provide no fitness advantage at all, rather being maintained by the putative toxin-antitoxin *hipAB* pair. Notably, we have observed expression of multiple genes on the plasmid in RNA-seq experiments (Fadeev et al., manuscript in preparation), suggesting that at least some of the genes are functional.

Plasmids and other extra-chromosomal elements can be identified experimentally (e.g., using PFGE, **Supplementary Figure S1**, López-Pérez et al., 2012), however, these methods require specific equipment, and are often not performed without initial evidence of such elements. Such evidence was provided by the efforts we put into obtaining a high-quality, fully sequenced and closed genome. Nevertheless, many of the draft assemblies could also have been useful, for example to provide reference genomes for transcriptomic analyses. Furthermore, with the high coverage obtained using Illumina sequencing, the *de novo* assemblies contained almost all of the coding genes (**Figure 3A**). Thus, with the appropriate caveats, a comparison of the functional capacities of the different *Alteromonas* strains could have been performed using a draft genome. Finally, phylogenetic analysis of the draft genome might have indicated horizontal transfer of some genes, yet only the revelation of a plasmid during genome closing shows a mechanism for horizontal transfer and identifies the transferrable contents. The reference-based and hybrid assemblies, in contrast, lack a significant part of the genomes and thus both comparative genomic and transcriptomic assays will clearly be biased, unless additional measures are taken to close the genome, e.g., through the use of complimentary methods such as optical mapping or gap-closing by PCR (e.g., López-Pérez et al., 2013).

## Author Contributions

EF and DS designed research. EF, DA, and DS isolated the DNA, produced in-house sequencing libraries and performed PFGE. EF, FP, AV, and SH assembled the genome, EF and DS analyzed the data. All authors participated in writing the manuscript.

## Conflict of Interest Statement

The authors declare that the research was conducted in the absence of any commercial or financial relationships that could be construed as a potential conflict of interest.

## Supplementary Material

FIGURE S1 **Validation of the presence of plasmid pAM1A3 using PFGE**. **(A)** Schematic illustration of the plasmid ring with the restriction digest sites; the table within the ring refers to the expected band sizes using each one of the enzymes. Two regions with higher sequencing coverage, which might be duplicated (see below), are shown. **(B)** PFGE gel of the plasmid isolated using NucleoBond BAC 100 kit, either uncut or after digesting with the three restriction enzymes shown in **(A)**. The ladder markers refer to a low range ladder PFGE marker, a lambda ladder PFGE and a 1 KB DNA ladder, with the sizes of the markers on the right of the gel image. **(C)** Inset showing the doubled bands observed when pAM1A3 was digested by two of the restriction enzymes. **(D)** Read coverage of the plasmid (from the Illumina HiSeq2000 library, see **Table 1**). “Hot spots” for single nucleotide polymorphisms (SNPs) are highlighted. The numbers below marked regions refer to the number of SNP’s in that region.

FIGURE S2 **Phylogenetic analysis of *Alteromonas* strains.** Maximum Likelihood tree of all *Alteromonas* strains for which a whole genome sequence is available. The numbers on the branches represent bootstraps percentage. **(A)** According to 16S rDNA sequences. **(B)** According to concatenated alignment of the following housekeeping genes: adenylylsulfate kinase, DNA adenine methylase, *dnaK*, *flgG*, *gyrB*, lipoyl synthase, malate dehydrogenase, octanoyltransferase, phosphoglucomutase, porphobilinogen deaminase, sulfate adenylyltransferase subunit 2.

FIGURE S3 **Abundance of the 100 most abundant functional SEED subsystems representation in different assembly methods compared to the final genome and plasmid.** Asterisk marked columns showed significant difference (*p* = 0.05, Fishers Exact Test with Benjamini-Hochberg False Discovery Rate).

FIGURE S4 **Sequence comparison using BLAST ring image generator (Alikhan et al., 2011), of pAM1A3 to four isolated *A. macleodii* plasmids and pGLAAG01 isolated from *Glaciecola sp.* 4H-3-7 + YE-5 (outer rings, colored according to the legend) to pAM1A3 (inner black thin ring).** The regions in the figure represent at least 70% identity and an *e*-value ≤ 0.00001.

FIGURE S5 **Pairwise alignment of region 1 (see **Figure 2**) of metal resistance fGI and pAM1A3.** The large gap in the alignment refers to a mobile element which is present in the fGI but absent in the pAM1A3 sequence. The alignment was conducted using Geneious aligner with default parameters.

FIGURE S6 **Phylogeny of selected genes on the pAM1A3 plasmid.** The gray rectangles refer to genes placed within the metal resistance fGI. The phylogenetic trees were built using the Neighbor–Joining method, with bootstrap representation of >80% in all of the branches in all of the trees. The numbers in round brackets refer to the position of the gene in the plasmid sequence.

FIGURE S7 **BlastN results of draft *A. macleodii* MIT1002 genome search against the sequence of pAM1A3 plasmid (*e*-value ≤10e-1).** Regions 1 and 2 refer to the mutual regions of the pAM1A3 plasmid and metal resistance fGI (see **Figure 2**).
