# Supplementary figures and images for: Why Close a Bacterial Genome? The Plasmid of Alteromonas Macleodii HOT1A3 is a Vector for Inter-Specific Transfer of a Flexible Genomic Island
Source: Front Microbiol. 2016 Mar 8;7:248. doi: 10.3389/fmicb.2016.00248 (PMC4781885; doi:10.3389/fmicb.2016.00248)

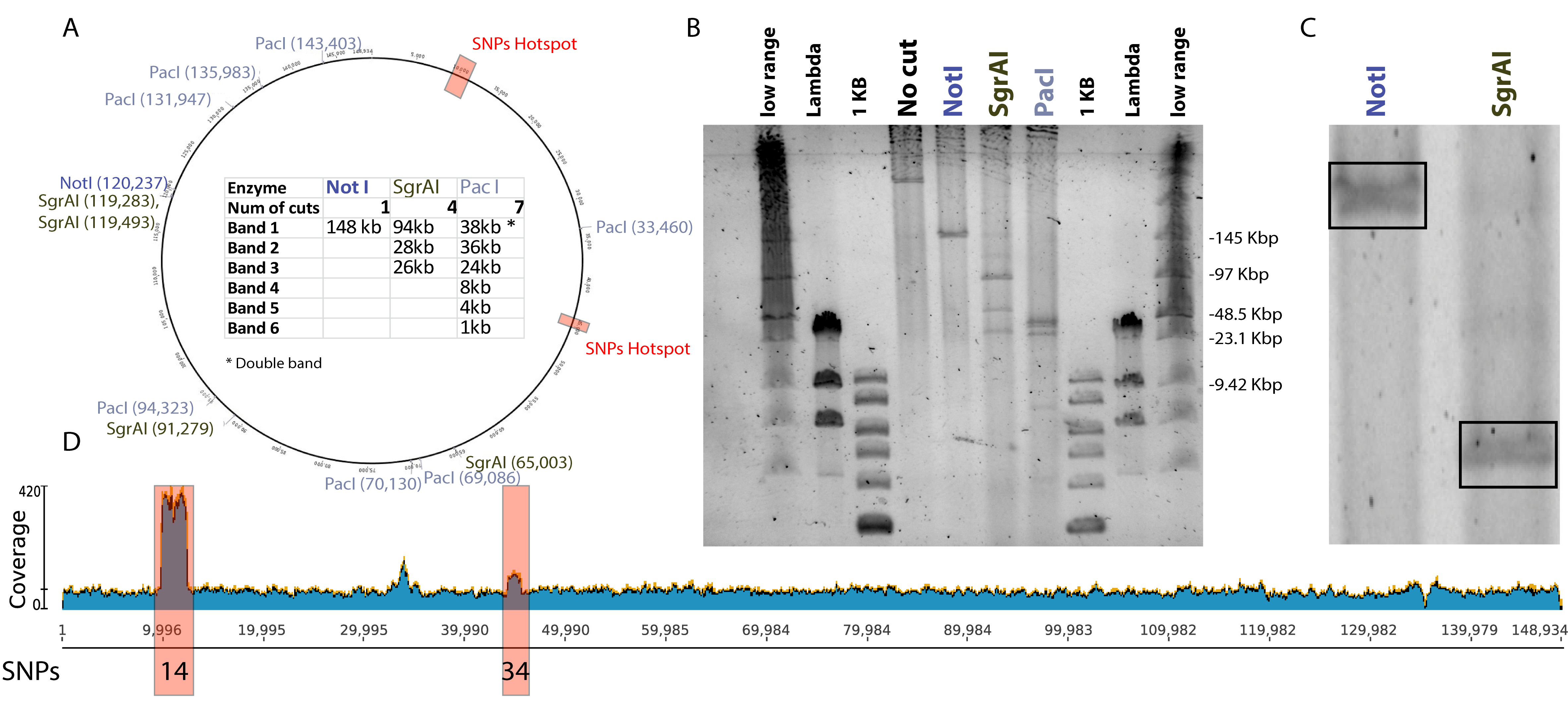

Supplement: FIGURE S1 — Validation of the presence of plasmid pAM1A3 using PFGE. (A) Schematic illustration of the plasmid ring with the restriction digest sites; the table within the ring refers to the expected band sizes using each one of the enzymes. Two regions with higher sequencing coverage, which might be duplicated (see below), are shown. (B) PFGE gel of the plasmid isolated using NucleoBond BAC 100 kit, either uncut or after digesting with the three restriction enzymes shown in (A). The ladder markers refer to a low range ladder PFGE marker, a lambda ladder PFGE and a 1 KB DNA ladder, with the sizes of the markers on the right of the gel image. (C) Inset showing the doubled bands observed when pAM1A3 was digested by two of the restriction enzymes. (D) Read coverage of the plasmid (from the Illumina HiSeq2000 library, see Table 1). “Hot spots” for single nucleotide polymorphisms (SNPs) are highlighted. The numbers below marked regions refer to the number of SNP’s in that region. [file Image_1.TIF]

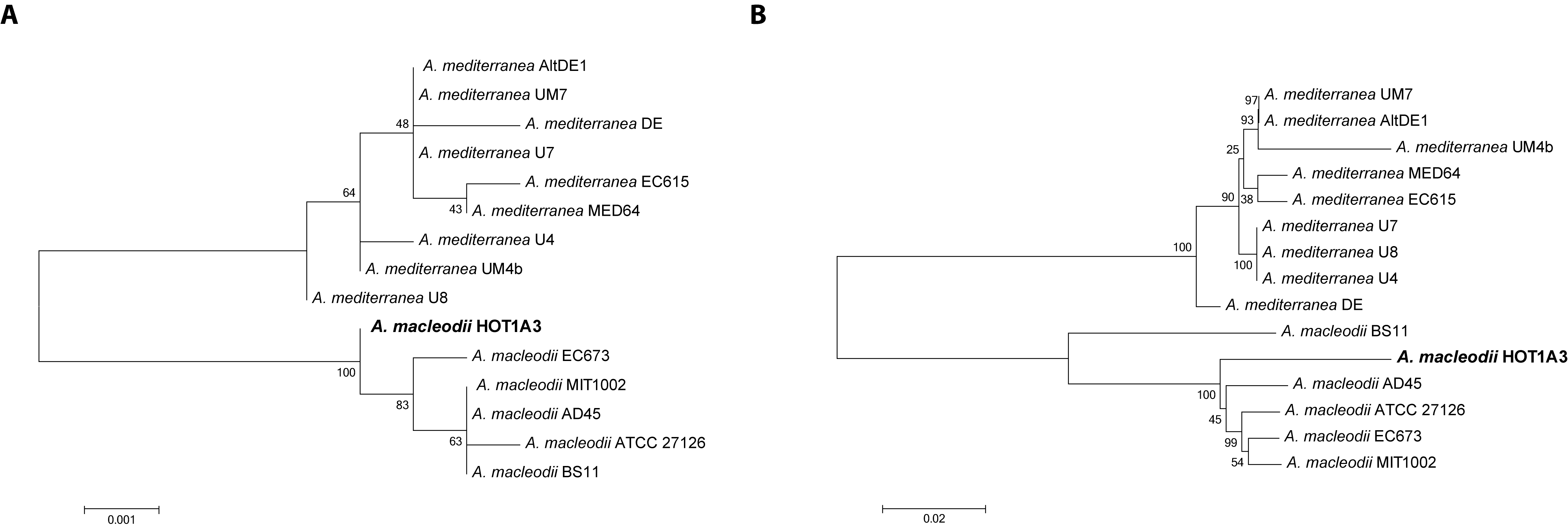

Supplement: FIGURE S2 — Phylogenetic analysis of Alteromonas strains. Maximum Likelihood tree of all Alteromonas strains for which a whole genome sequence is available. The numbers on the branches represent bootstraps percentage. (A) According to 16S rDNA sequences. (B) According to concatenated alignment of the following housekeeping genes: adenylylsulfate kinase, DNA adenine methylase, dnaK, flgG, gyrB, lipoyl synthase, malate dehydrogenase, octanoyltransferase, phosphoglucomutase, porphobilinogen deaminase, sulfate adenylyltransferase subunit 2. [file Image_2.TIF]

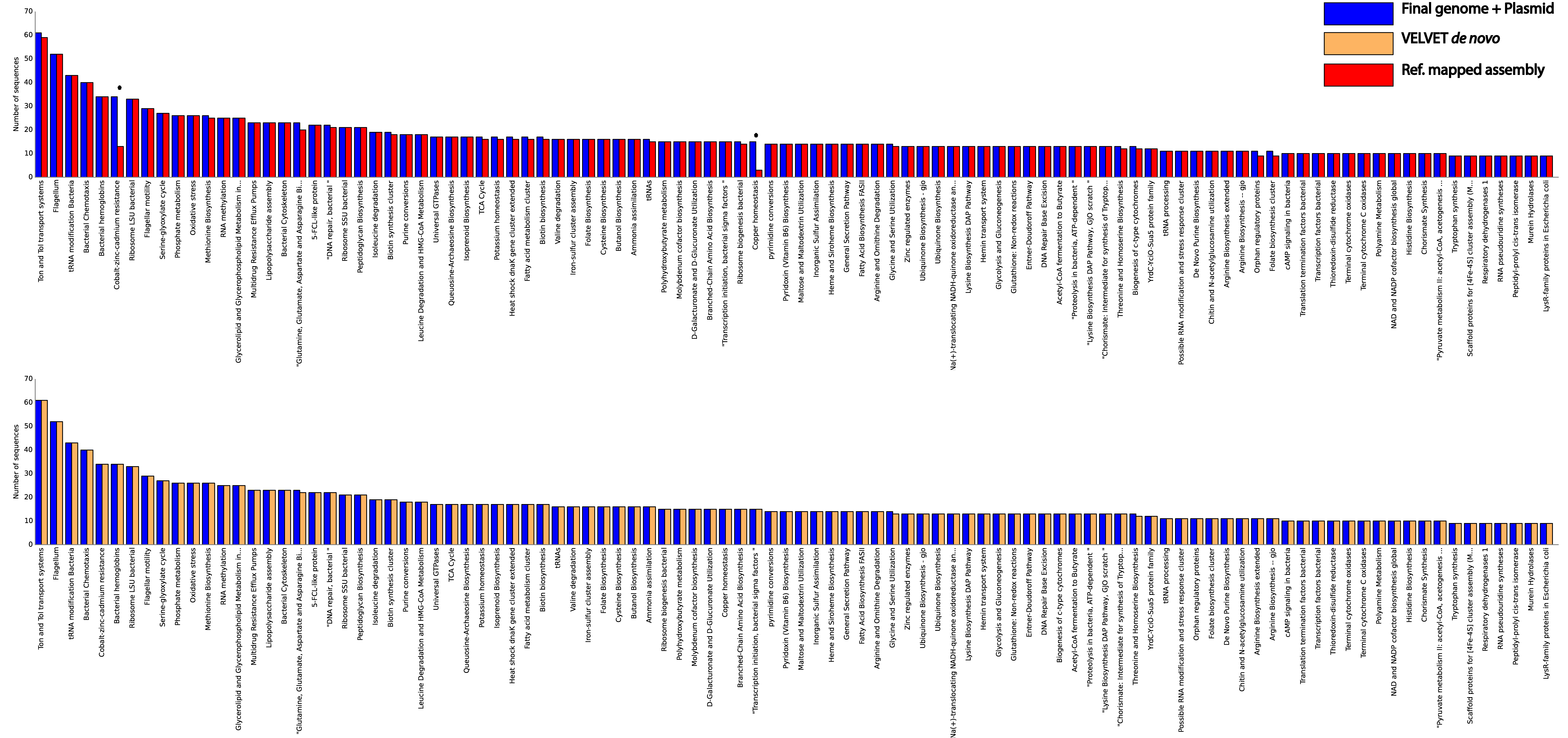

Supplement: FIGURE S3 — Abundance of the 100 most abundant functional SEED subsystems representation in different assembly methods compared to the final genome and plasmid. Asterisk marked columns showed significant difference (p = 0.05, Fishers Exact Test with Benjamini-Hochberg False Discovery Rate). [file Image_3.TIF]

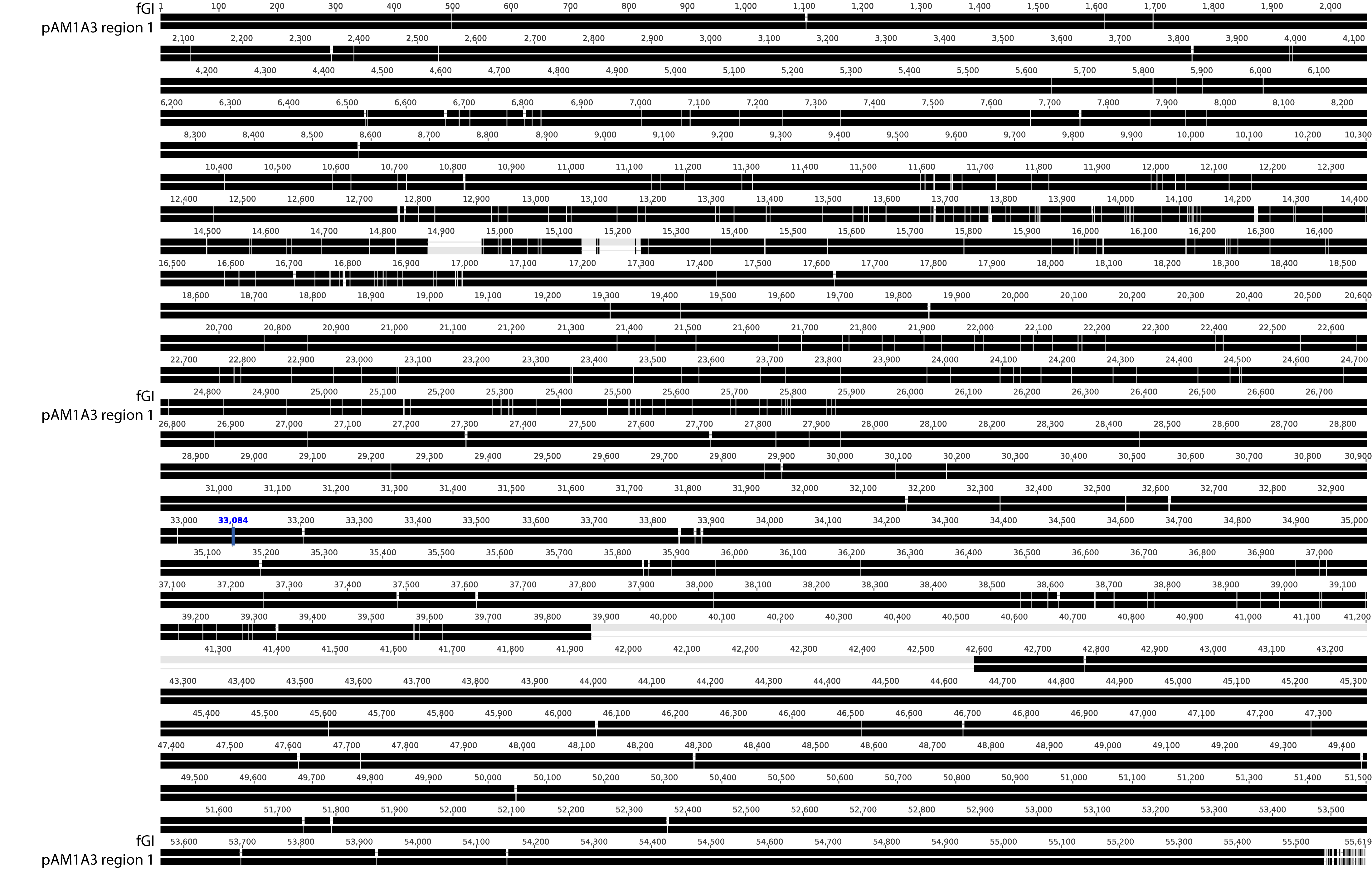

Supplement: FIGURE S5 — Pairwise alignment of region 1 (see Figure 2) of metal resistance fGI and pAM1A3. The large gap in the alignment refers to a mobile element which is present in the fGI but absent in the pAM1A3 sequence. The alignment was conducted using Geneious aligner with default parameters. [file Image_5.TIF]

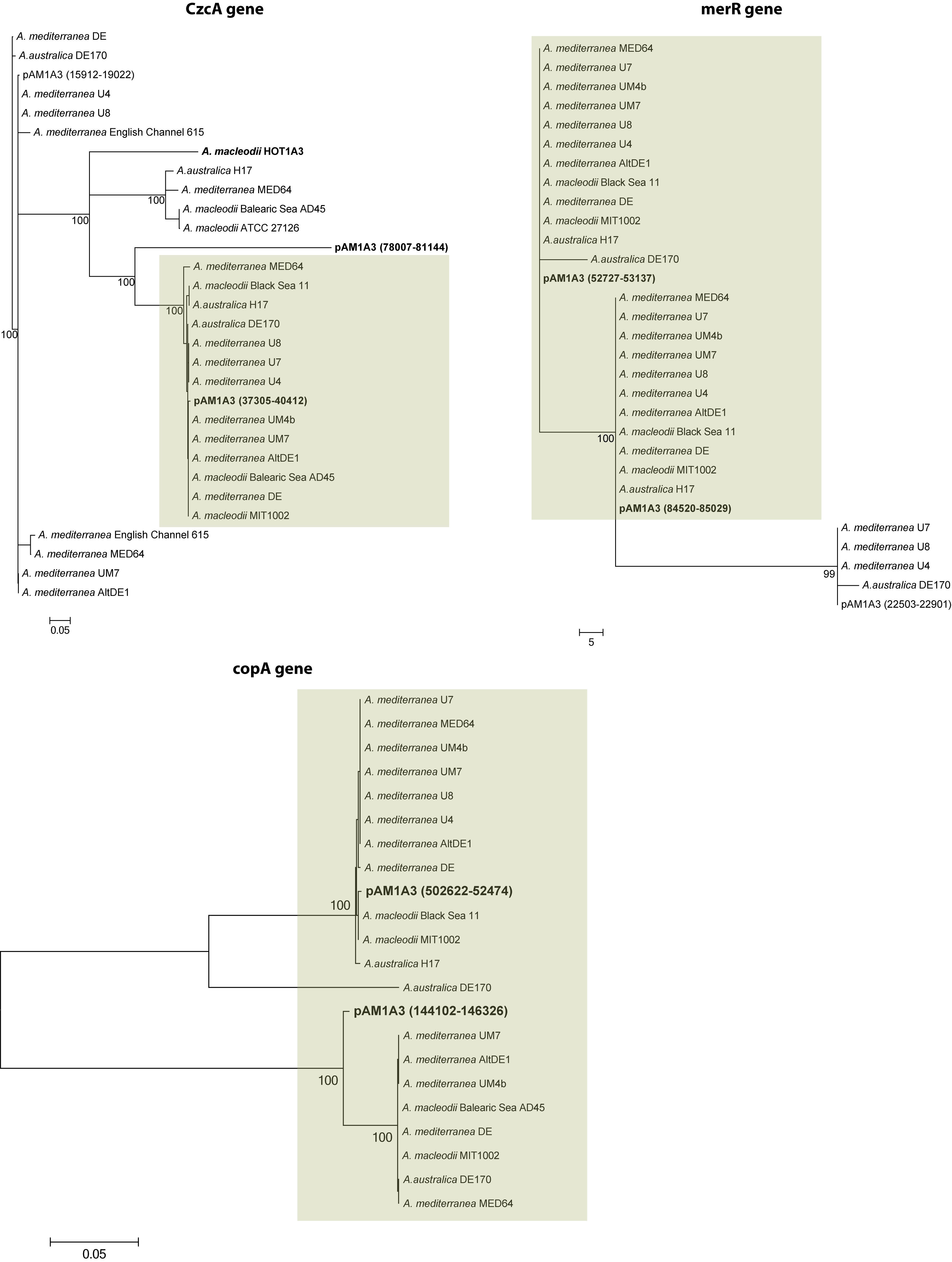

Supplement: FIGURE S6 — Phylogeny of selected genes on the pAM1A3 plasmid. The gray rectangles refer to genes placed within the metal resistance fGI. The phylogenetic trees were built using the Neighbor–Joining method, with bootstrap representation of >80% in all of the branches in all of the trees. The numbers in round brackets refer to the position of the gene in the plasmid sequence. [file Image_6.TIF]

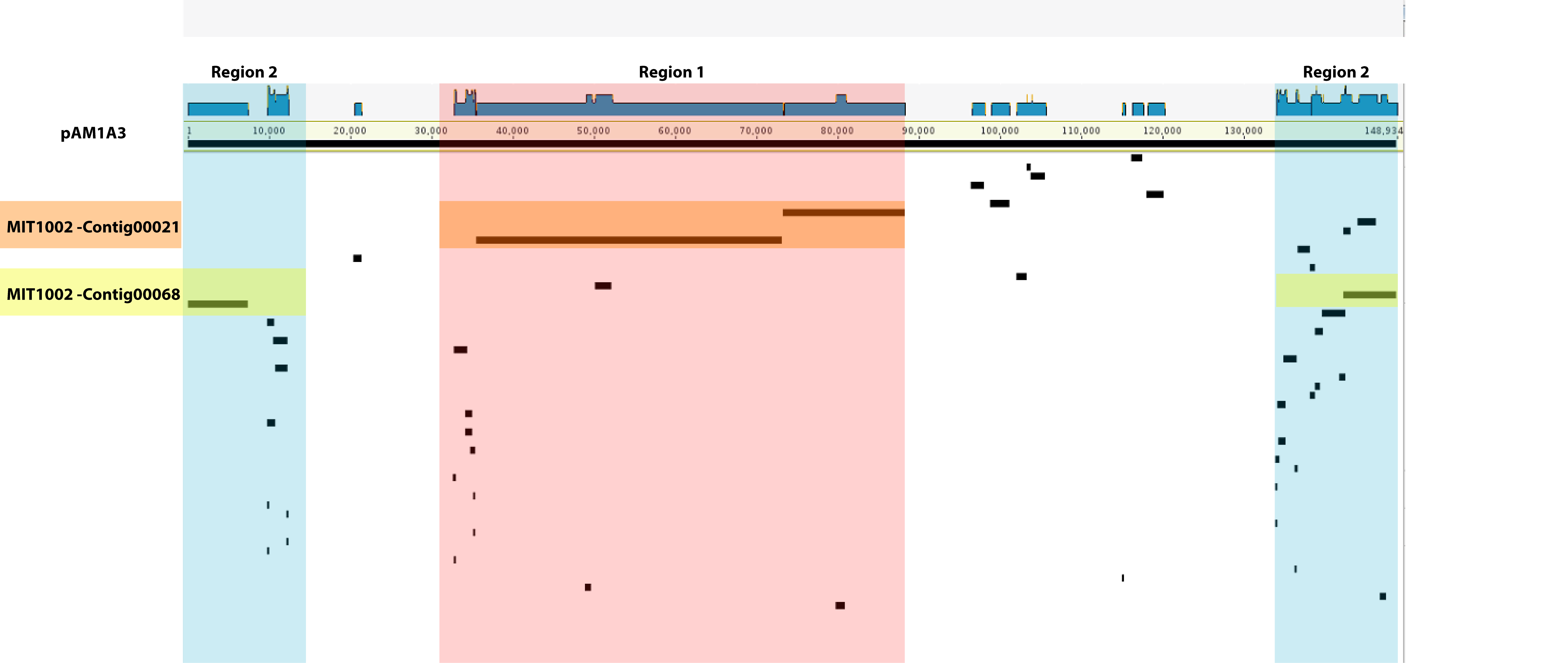

Supplement: FIGURE S7 — BlastN results of draft A. macleodii MIT1002 genome search against the sequence of pAM1A3 plasmid (e-value ≤10e-1). Regions 1 and 2 refer to the mutual regions of the pAM1A3 plasmid and metal resistance fGI (see Figure 2). [file Image_7.TIF]
